# *Diaph1* knockout inhibits mouse primordial germ cell proliferation and affects gonadal development

**DOI:** 10.1186/s12958-024-01257-z

**Published:** 2024-07-15

**Authors:** Xin Zhao, Chunbiao Fan, Tongtong Qie, Xinrui Fu, Xiaoshuang Chen, Yujia Wang, Yuan Wu, Xinyao Fu, Kesong Shi, Wenlong Yan, Haiquan Yu

**Affiliations:** 1 School of Biology and Agriculture, Shaoguan University, Shaoguan, 512005 Guangdong Province China; 2 State Key Laboratory of Reproductive Regulation and Breeding of Grassland Livestock, School of Life Sciences, Inner Mongolia University, Hohhot, 010020 Inner Mongolia China

**Keywords:** Diaph1, Primordial germ cells, Migration, Leydig cells, Granulosa cells, Gonadal development

## Abstract

**Background:**

Exploring the molecular mechanisms of primordial germ cell (PGC) migration and the involvement of gonadal somatic cells in gonad development is valuable for comprehending the origins and potential treatments of reproductive-related diseases.

**Methods:**

Diaphanous related formin 1 (*Diaph1, also known as mDia1*) was screened by analyzing publicly available datasets (ATAC-seq, DNase-seq, and RNA-seq). Subsequently, the CRISPR-Cas9 technology was used to construct *Diaph1* knockout mice to investigate the role of *Diaph1* in gonad development.

**Results:**

Based on data from public databases, a differentially expressed gene *Diaph1*, was identified in the migration of mouse PGC. Additionally, the number of PGCs was significantly reduced in *Diaph1* knockout mice compared to wild type mice, and the expression levels of genes related to proliferation (*Dicer1*, *Mcm9*), adhesion (*E-cadherin*, *Cdh1*), and migration (*Cxcr4*, *Hmgcr*, *Daz*l) were significantly decreased. *Diaph1* knockout also inhibited Leydig cell proliferation and induced apoptosis in the testis, as well as granulosa cell apoptosis in the ovary. Moreover, the sperm count in the epididymal region and the count of ovarian follicles were significantly reduced in Diaph1 knockout mice, resulting in decreased fertility, concomitant with lowered levels of serum testosterone and estradiol. Further research found that in *Diaph1* knockout mice, the key enzymes involved in testosterone synthesis (CYP11A1, 3β-HSD) were decreased in Leydig cells, and the estradiol-associated factor (FSH receptor, AMH) in granulosa cells were also downregulated.

**Conclusions:**

Overall, our findings indicate that the knockout of *Diaph1* can disrupt the expression of factors that regulate sex hormone production, leading to impaired secretion of sex hormones, ultimately resulting in damage to reproductive function. These results provide a new perspective on the molecular mechanisms underlying PGC migration and gonadal development, and offer valuable insights for further research on the causes, diagnosis, and treatment of related diseases.

**Supplementary Information:**

The online version contains supplementary material available at 10.1186/s12958-024-01257-z.

## Background

In the mouse, primordial germ cells (PGCs) initially appear at the base of the incipient allantois at embryonic day 6.5 (E6.5). Subsequently, at E9.5, PGCs rapidly traverse from the basal lamina of the gut epithelium into the mesentery, and ultimately colonize the genital ridges at E11.5 [1, 2]. The migration of PGCs primarily depends on the regulation of adhesion to neighboring cells and the extracellular matrix [3, 4]. PGC migration is governed by various mechanisms, including the signaling pathways involving Steel and c-Kit [5], Wnt5a and Ror2 [6, 7], and the chemokine Sdf1/Cxcr4 pathway [8]. Furthermore, PGCs proliferate actively during the migratory phase, the number of PGCs increases drastically from ∼40 at E7.25 to ∼25,000 at E13.5 [9].

After reaching the genital ridges, the PGCs aggregate, lose migratory ability, and are surrounded by somatic cells of the genital ridges, forming mature gametes [10]. The proper development of both the gonadal somatic cells and germ cells is crucial for maintaining gonadal function and facilitating gametogenesis, this process is regulated by a complex and coordinated molecular program, which ensures the accurate differentiation of reproductive cell types and the sustained functionality of these cells into adulthood [11]. In males, testicular stromal cells, as crucial members of the testicular cells, play a vital role in producing testosterone, which is essential for supporting sperm production in adulthood [12–14]. Insufficient quantities or dysfunction of Leydig cells can lead to testosterone deficiency, which in turn can cause disorders related to androgen deficiency in males [15, 16]. In female, the granulosa cells of follicles play a determining role in ovarian development [17], including the synthesis and secretion of steroid hormones, such as estradiol (E2) and progesterone [18, 19]. These hormones participate in the regulation of follicle development at different stages, providing support for the maturation of oocytes [20].

Recent studies have made use of publicly available databases to analyze chromatin properties and gene expression patterns in order to gain insights into the intricate processes involved in reproductive development [21–23]. Among them, our research group previously utilized publicly available chromatin property data and gene expression data to construct a transcription factor-mediated gene regulatory network during the formation of spermatogonia stem cells, and identified key spermatogonia stem cell-specific transcription factors and genes [23]. In this study, based on multi-omics data, we screened functional molecules involved in the migration of PGC. Among these molecules, we specifically investigated the role of the differentially expressed gene *Diaph1* (*also known as mDia1*), a member of the formin family. Formin family proteins were reported to be associated with cellular and molecular functions such as migration, contraction, adhesion, cell division, and microtubule regulation [24, 25]. To explore the significance of *Diaph1* in PGC migration and gonadal development, we constructed *Diaph1* knockout mice and investigated its impact on these processes.

## Methods

### Data sources and bioinformatics analysis

The chromatin property DNase-seq data and RNA-seq data of E9.5/E10.5/E12.5 PGCs were derived from the GSE109767 and GSE94136 dataset, respectively. In our previous studies [23], we provided detailed explanations of the complex data processing methods used for DNase-seq and RNA-seq. In brief, raw sequences for DNase-seq and RNA-seq data were processed by Cutadapt v1.9. Then, the reads were aligned to the reference genome (mm10) using Bowtie2 v2.4.4 or HISAT2 v2.2.1. Differential peak analysis and differential gene analysis was performed using DiffBind v3.4.0 and limma v3.48.3, respectively. Moreover, the functional annotation and pathway enrichment of the differentially expressed genes were conducted using DAVID (*FDR* < 0.05) [26].

### Animal

The *Diaph1* knockout mice in this study were generated using the CRISPR-Cas9 technique and obtained from Cyagen Biosciences (Su Zhou, China). A schematic experimental design illustrating the *Diaph1* knockout in mice can be found in Additional file 1: Fig. S1A. The wild-type allele had an amplicon of 8294 bp, while the Diaph1 + / − heterozygous mice allele had amplicons of 898 bp/335 bp/692 bp. The Diaph1-/- homozygous allele contained an amplicon of 898 bp/335 bp. The primer sequences used for identifying the genotype of Diaph1 knockout mice can be found in Additional file 2: Table 1. Specific-pathogen-free (SPF) C57Bl6/J wild-type mice were obtained from Animal Center of Inner Mongolia University. Additionally, Oct4-GFP mice were also obtained from Cyagen Biosciences (Su Zhou, China). All mice were reared at the Animal Center of Inner Mongolia University, and all experiments were approved by the Animal Care and Use Committee of Inner Mongolia University (approval ID: IMU-2020-mouse -043).

### Acquisition of mouse primordial germ cells, testicular Leydig and ovarian granulosa cells

Embryonic fragments containing PGCs (at E9.5/E10.5) or genital ridges (at E12.5) were trypsinized and sorted using fluorescence-activated cell sorting (FACS) with OCT-4-GFP as a marker. Testes and ovaries were collected from mice, and fat surrounding the testis and ovary was removed. Both Leydig cells and granulosa cells were sorted using FACS. The cultured Leydig cells and granulosa cells were maintained in DMEM/F-12 (VivaCell, Shanghai, China) supplemented with 15% FBS.

### Fluorescence-activated cell sorting analysis (FACS)

Mouse embryonic, testis, and ovary tissues were digested using pancreatin. The resulting single-cell suspension was then rinsed with PBS and fixed with 4% paraformaldehyde in PBS at room temperature for 20 min. After two washes with PBS, the cells were incubated with a mixture of primary antibodies on ice for 60 min. Following another two washes with PBS, the cells were incubated with the secondary antibody for 60 min. PGCs expressing OCT4-ΔPE-GFP were isolated from embryos using FACS, and no primary antibodies were needed for this process. FACS was performed using BD SORP ARIA III (BD Biosciences).

### Immunofluorescence and immunohistochemistry of paraffin sections

Immunohistochemistry staining was performed on paraffin sections obtained from embryos at different stages of development. The sections were initially incubated at room temperature for 1 h in a 5% bovine serum albumin (BSA) solution. Subsequently, the sections were incubated overnight at 4 °C with primary antibodies anti-OCT4 (1:400, ab27985, Abcam, Cambridge, UK) and 3β-HSD (1:400, ab65156, Abcam, Cambridge, UK). The next day, secondary antibodies were applied to the sections for immunolabeling of syntropies. Immunofluorescence images were captured using a fluorescence microscope (Nikon laser confocal microscope, Nikon, Japan). For immunohistochemistry, paraffin-embedded sections from ovaries and testis were utilized. The slides were deparaffinized and washed three times with ethanol, using a graded series of concentrations (100%, 85%, 75%). Following a 5-min rinse in distilled water, the slides were dewaxed and treated with citric acid antigen repair buffer to repair the antigen. Next, the section was blocked for 1 h using a 3% BSA solution, and then incubated overnight at room temperature with the primary antibody. After washing with PBST, the slides were incubated for 2 h at room temperature with the secondary antibodies (1:2000, Invitrogen, USA). Finally, the slides were rinsed with PBST and counterstained using DAPI.

### Cell immunofluorescence staining and AP staining

The cultured cells were washed three times with PBS and subsequently fixed with 4% polyoxymethylene for 20 min. Next, the cells were permeabilized using a 0.5% Triton-X100 solution, followed by three washes with PBS. To block any nonspecific binding, the cells were treated with 5% BSA for 60 min. Afterward, the cells were incubated overnight at 4 °C with primary antibodies. The following day, the cells were washed three times with PBS for 5 min each and then incubated with the secondary antibody for 90 min at room temperature. Finally, DAPI was used for counterstaining, and the cells were photographed using a Nikon laser confocal microscope (Nikon, Japan). For AP staining, the cells were fixed using 4% paraformaldehyde for 20 min and then washed three times with PBS. Subsequently, an AP detection kit (SCR004, Sigma) was employed to detect AP activity, in accordance with the instructions provided by the manufacturer.

### Flow cytometry

The cell cycle was analyzed using flow cytometry. Leydig cells and granulosa cells (5 × 10^5^ cells/well) were seeded in a 6-well plate at a concentration of 1 × 10^5^ cells/mL and allowed to adhere overnight. The cells were then collected and washed twice with cold 1 × PBS. Afterward, they were resuspended in 1 mL of 70% ethanol and stored overnight. Following this, the cells were washed again with cold PBS and stained with 500 μL of PI staining solution for 30 min at 37 °C in the dark. To analyze the cell cycle, a cell cycle analysis kit (BA00205, Bioss, China) was used, and the percentage of cells in each stage of the cell cycle was determined using FlowJo_v10.6.2 software. Cell apoptosis was evaluated by flow cytometry. The cells were collected in conical tubes and washed twice with cold PBS. The suspension (5 × 10^5^ cells/well) in binding buffer was then treated with the Annexin V-FITC/PI cell apoptosis detection kit (FA101-01, Beijing, China) at room temperature for 30 min. Subsequently, the cells were analyzed using flow cytometry (Beckman Coulter).

### RNA isolation, qRT‒PCR, and Western blot analysis

Total RNA was extracted from cells using RNeasy reagents TRIzol reagent (Invitrogen, USA). The RNA is then converted into complementary DNA (cDNA) through reverse transcription using the Reverse Transcription kit (RR047A, Takara, Japan) following the instructions provided by the manufacturer. Then, qRT‒PCR was performed using an ABI 7500 instrument (Applied Biosystems, Foster City, CA, USA) with GoTaq® qPCR Master Mix (Promega, Wisconsin, USA) according to the manufacturer’s protocols. The primer sequences are provided in Additional file 2: Table 2.

For western blot, the proteins were isolated with RIPA lysis buffer (R0010, Solarbio, China). The protein concentration was measured by Pierce BCA Protein Assay Kit (23,227, Thermofisher). 30 µg of protein was separated by 10% SDS-PAGE and then transferred onto PVDF (0.45 μm) membranes (IPVH00010, Immobilon-P, Millipore). The membranes were blocked with 5% skimmed milk at room temperature for 1 h. The primary antibodies (1:1000) were incubated with the membranes overnight at 4 °C. The next day, the membranes were incubated with secondary antibodies (SA00001-1 or SA00001-2, Proteintech) at room temperature for 1 h, followed by detection using the chemiluminescence Tanon-5200 (Tanon, Shanghai, China). Western blotting was performed using the following specific antibodies: OCT4 (ab27985, Abcam, Cambridge, UK), 3β-HSD (ab65156, Abcam, Cambridge, UK); DIAPH1 (20,624–1-AP), CYP11A1 (13,363–1-AP), GAPDH (60,004–1-Ig), BCL-2 (26,593–1-AP), BAX (50,599–2-Ig), and P53 (60,283–2-Ig) obtained from Proteintech Biotechnology (Wuhan, China). All the primary antibodies were utilized at a dilution of 1:1,000, and the secondary antibodies at a dilution between 1:2,000. The original pictures of western blots are listed in Additional file 3.

### ELISA assays

Leydig cell and granulosa cells were seeded into 6 well plates at a density of 5.0 × 10^5^ cells/well with DMEM/F-12 (VivaCell, Shanghai, China)) with 15% FBS. After incubation overnight, the culture medium was then collected, centrifuged to remove the dead cells and debris, and the supernatants were subjected to ELISA analysis. The concentrations of follicle-stimulating hormone (FSH), luteinizing hormone (LH), and testosterone were measured using specific enzyme-linked immunosorbent assay (ELISA) kits designed for mice. The FSH assay employed the mouse FSH ELISA kit (EM1035), the LH assay used the mouse LH ELISA kit (EM1188), and the testosterone levels were determined with the mouse testosterone ELISA kit (EM1850). These ELISA kits were sourced from Fine Test Company in Hubei, China.

### CCK8 assay

Cell proliferation was evaluated through the CCK-8 assay. Specifically, cells were seeded at a density of 1 × 10^3^ cells per well on 96-well plates and then exposed to 10 μl of CCK-8 reagent (TransGen, Beijing, China). The cell counts were conducted every 24 h over a span of three days.

### Determination of sperm motility

The total number of motile spermatozoa (approximately 200 cells) was assessed by two independent observers using phase-contrast microscopy (Olympus Corporation, Japan), and the percentage of forward motility was subsequently calculated.

### Statistical analysis

The data is presented as the mean ± standard deviation. Additionally, significant differences were evaluated using two-tailed Student's t-test or one-way ANOVA for comparisons between multiple groups in GraphPad Prism 7.0 (GraphPad Software, La Jolla, United States). A Tukey test was used for post hoc pairwise comparisons. For statistical significance, * denotes *p value* < 0.05, ** denotes *p value* < 0.01, and *** denotes *p value* < 0.001.

## Results

### Screening of mouse PGCs migration-related genes

**Fig. 1:**
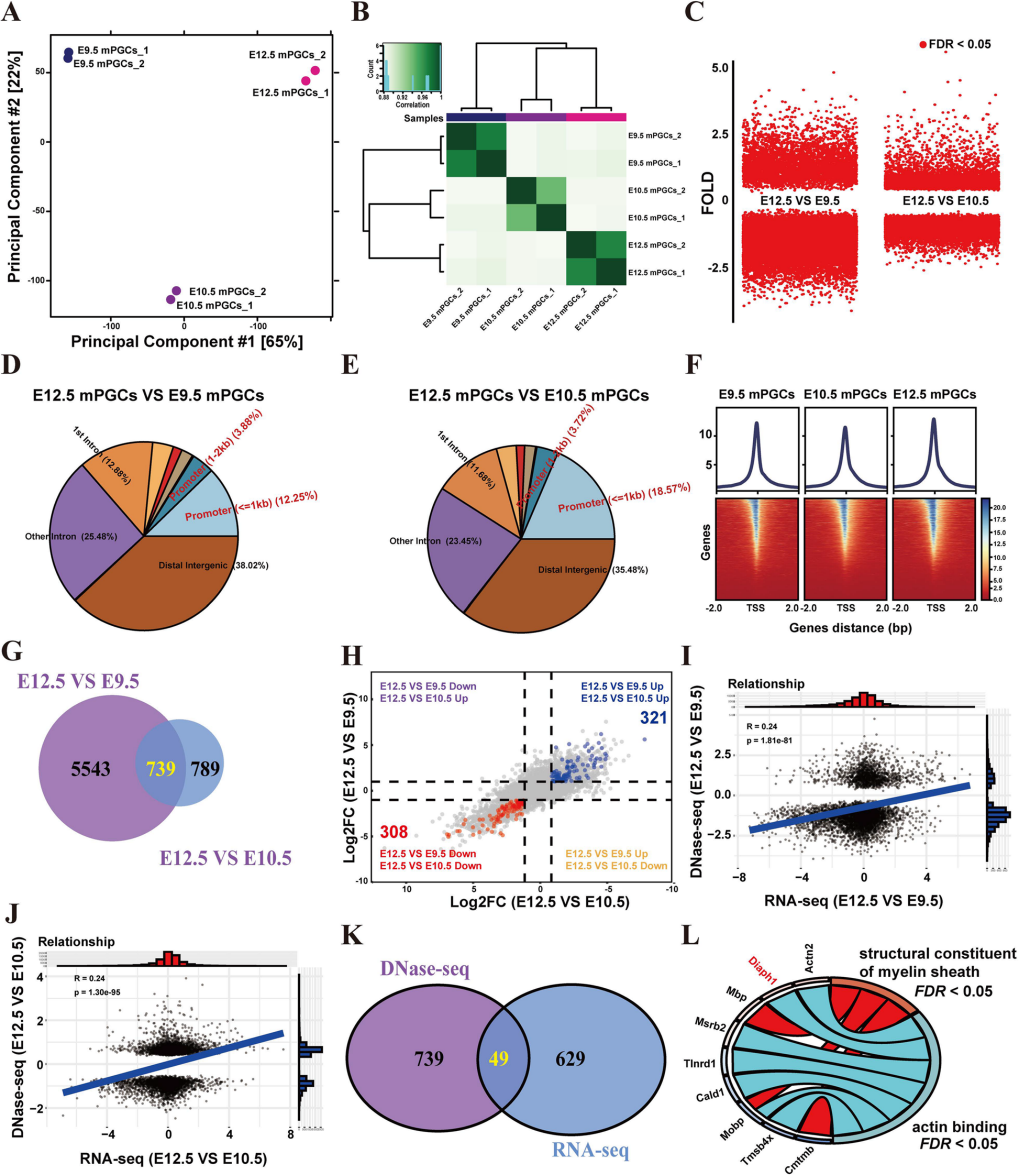
Screening of mouse PGC migration-related genes. **A**, **B** Principal component analysis (**A**) and correlation analysis (**B**) of the DNase-seq data between E9.5, E10.5, and E12.5 mPGCs. **C** Volcano plot for differential peak expression between E9.5, E10.5, and E12.5 mPGCs. **D**, **E** Distribution of differential DNase-seq peaks of E12.5 VS E9.5 mPGCs (**D**) and E12.5 VS E10.5 mPGCs (**E**). **F** Line plot shows the chromatin accessibility in E9.5, E10.5, and E12.5 mPGCs around the TSS. **G** Venn diagram showing numbers of overlapping differentially expressed annotated genes. **H** Dot plot of differential genes. **I**, **J** Point density plots indicating the correlation between differential peak expression (DNase-seq) and differential gene expression levels (RNA-seq) in E12.5 VS E9.5 mPGCs (**I**) and E12.5 VS E10.5 mPGCs (**J**). **K** Venn diagram showing numbers of overlapping genes. **L** GO biological process analysis of overlapping genes

Considering the complexity of gene expression regulation during mouse PGC (mPGC) migration, a multi-omics approach becomes imperative for the identification of genes associated with mPGC migration. Therefore, we first conducted chromatin accessibility analysis on mPGCs at different developmental stages (E9.5, E10.5, and E12.5) based on previously published data. The analysis employed principal component analysis (PCA) (Fig. 1A) and hierarchical clustering analysis (Fig. 1B), revealing notable disparities in the expression patterns during PGC migration. The chromatin accessibility levels of 9218 accessible peaks (|fold change|> 1.5, false discovery rate *FDR* < 0.05) in E12.5 mPGCs were found to be altered compared to E9.5 PGCs (Fig. 1C). In addition, we identified 18,114 differentially accessible peaks in E12.5 mPGCs compared to E10.5 mPGCs (Fig. 1C). Afterwards, these differentially accessible peaks were annotated to the nearest gene. Among them, 16.13% and 22.29% of the binding sites are in promoter (the 2 kb region upstream and downstream), respectively (Fig. 1D-E). Additionally, a significant proportion of peaks were observed near the transcription start site (TSS) (Fig. 1F), and a total of 739 overlapping differentially expressed annotated genes were found (Fig. 1G).

Next, we conducted a comparison of the transcriptomic profiles between E9.5, E10.5, and E12.5 PGCs using publicly available RNA-seq data. Our analysis revealed that 2,294 genes exhibited differential expression between E12.5 and E9.5 PGCs, while 817 genes were differentially expressed between E12.5 and E10.5 PGCs (Fig. 1H). Moreover, a total of 629 genes were found to be shared between the differentially expressed genes (DEGs) in both the E12.5 vs E9.5 and E12.5 vs E10.5 PGCs (Fig. 1H). To clarify the functional relationships between chromatin property and RNA-seq data during the migration of PGCs, the relationship between gene expression and chromatin accessibility was analyzed. We found a significant correlation between open chromatin regions and gene expression in PGCs at E12.5 compared to E9.5 (correlation coefficient *R* = 0.24, *p value* = 1.81e-81), as well as in PGCs at E12.5 compared to E10.5 (*R* = 0.24, *p value* = 1.30e-95) (Fig. 1I-1J). Furthermore, we identified a total of 49 overlapping DEGs by comparing DEGs between DNase-seq and RNA-seq data (Fig. 1K). To further understand the functional roles of these overlapping DEGs during PGCs migration, Gene Ontology (GO) enrichment analysis was performed. GO analysis revealed that these overlapping DEGs were significantly associated with the structural constituent of myelin sheath and actin binding (Fig. 1L). These data indicate that overlapping DEGs may plays a role during PGCs migration.

### DIAPH1 was expressed differentially during mPGC migration

**Fig. 2:**
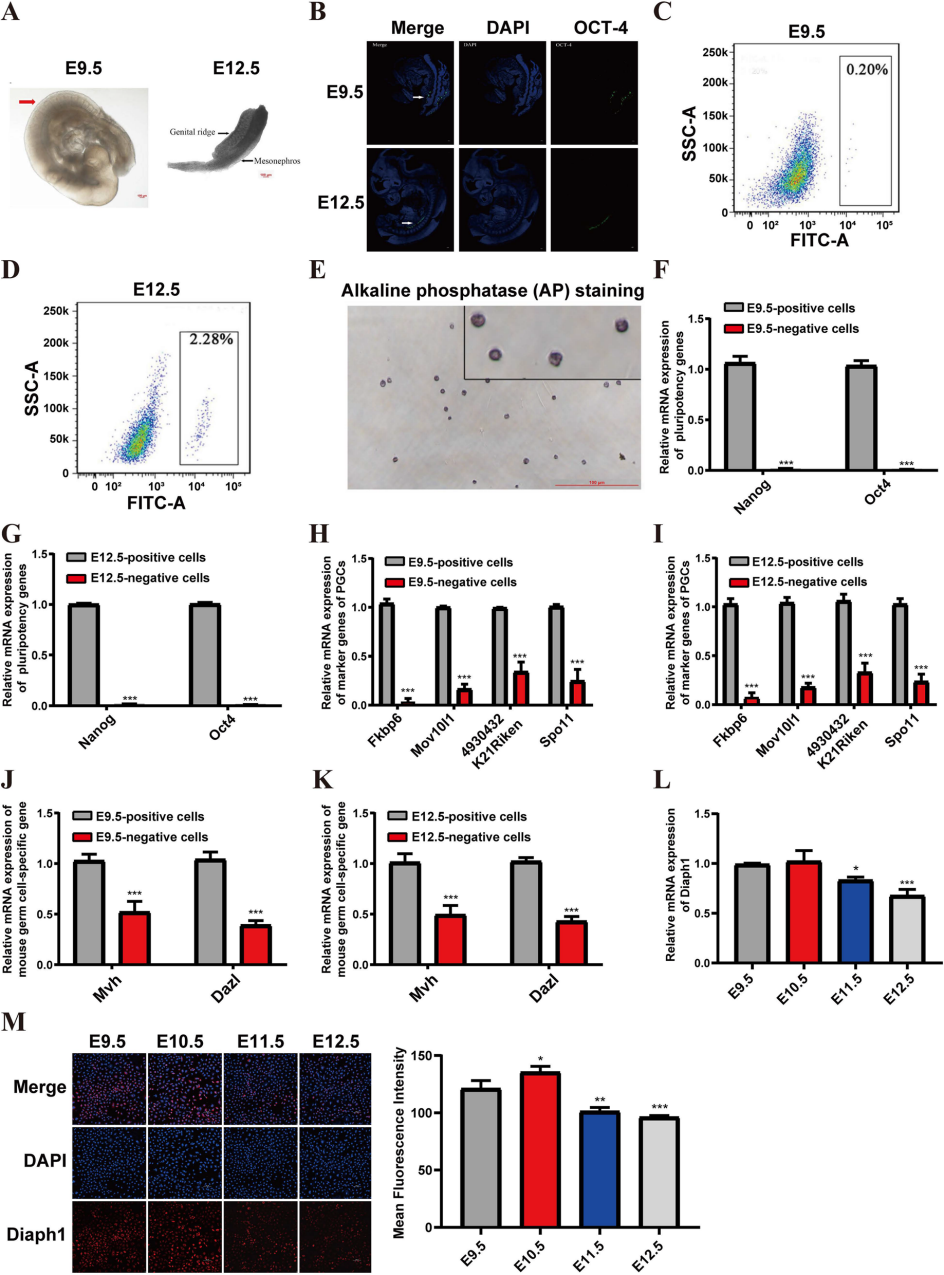
The acquisition and identification of mPGCs and validation of *Diaph1* expression. **A** Frontal view of E9.5 mouse embryos (left) and E12.5 genital ridges (right). The position of posterior third of E9.5 mouse embryos by arrowheads. Scale bar: 100 μm. **B** The localization of OCT4 at different embryonic stages (E9.5 and E12.5). **C**, **D** The mPGCs of E9.5 (**C**) and E12.5 (**D**) were isolated by FACS sorting for OCT4 positive cells. **E** Alkaline Phosphatase (AP) staining. **F**, **G** qRT-PCR for pluripotency marker genes at E9.5 (**F**) and E12.5 (**G**). **H**, **I** qRT-PCR for mPGCs marker genes at E9.5 (**H**) and E12.5 (**I**). **J**, **K** qRT-PCR for germ cell-specific genes at E9.5 (**J**) and E12.5 (**K**). **L** *Diaph1* mRNA expression was determined mPGCs by qRT-PCR in E9.5-E12.5

Based on the above results, overlapping DEGs were screened between DNase-seq and RNA-seq data during mPGC migration. Through the review of the literature, we found that *Diaph1*, was among the overlapping genes, play major roles in cell migration [27, 28]. However, its role in PGC migration has not been studied or reported. Hence, we selected *Diaph1* for subsequent experiments. To validate the differential expression of Diaph1 during mPGC migration, we first collected C57BL6/J mouse embryonic tissues at E9.5 and E12.5 mPGCs (Fig. 2A). Immunofluorescence staining was performed to detect the localization of the pluripotency and germ cell marker protein OCT4 in mouse embryonic tissues. The results revealed that at E9.5, OCT4 was localized in the posterior part of the embryo, while at E12.5, it was predominantly found in the genital ridge (Fig. 2B). Thus, these results demonstrate the utility of Oct4 as a marker for primordial germ cells. To isolate tissues containing fetal germ cells, we utilized transgenic mice carrying an Oct4 promoter-driven GFP reporter (OCT4-ΔPE-GFP). Tissues from different stages of embryos (posterior third of E9.5 embryos or genital ridges of E12.5 embryos) were collected and enzymatically dissociated to obtain single cell suspensions, which were then purified using fluorescence-activated cell sorting (FACS) based on the expression of the OCT4-GFP transgene (Fig. 2C-2D). The OCT4-positive cells were further confirmed by alkaline phosphatase staining (Fig. 2E). Moreover, the mRNA expression of pluripotency marker genes (*Nanog*, *Oct4*) and PGCs marker genes (*Fkbp6*, *Mov10l1*, *4930432K21Riken*, *Spo11*), and germ cell-specific genes (*Mvh*, *Dazl*) was assessed using qRT-PCR. The results demonstrated significantly higher expression of pluripotency marker genes (Fig. 2F-2G), PGCs marker genes (Fig. 2H-2I), and germ cell-specific genes (Fig. 2J-2K) was in OCT4-positive cells compared to OCT4-negative cells. This strongly supports that the OCT4-positive cells isolated by FACS are PGCs cells. Furthermore, the expression of the *Diaph1* gene in PGCs at different migration stages was validated via qRT-PCR. The results showed a significant downregulation of *Diaph1* expression in E11.5 and E12.5 PGCs compared to E9.5 PGCs (Fig. 2L). However, in comparison to E9.5 PGCs, the expression of *Diaph1* was higher at E10.5, although not significantly (Fig. 2L). Immunofluorescence analysis further confirmed a significant downregulation of DIAPH1 expression in E11.5 and E12.5 PGCs in contrast to E9.5 PGCs, whereas DIAPH1 expression at E10.5 was significantly higher than at E9.5 (Fig. 2M). These results indicate that DIAPH1 exhibits differential expression during PGC migration, suggesting its potential participation in the migration of mPGCs.

### Knocking out* Diaph1* inhibits PGC development

**Fig. 3:**
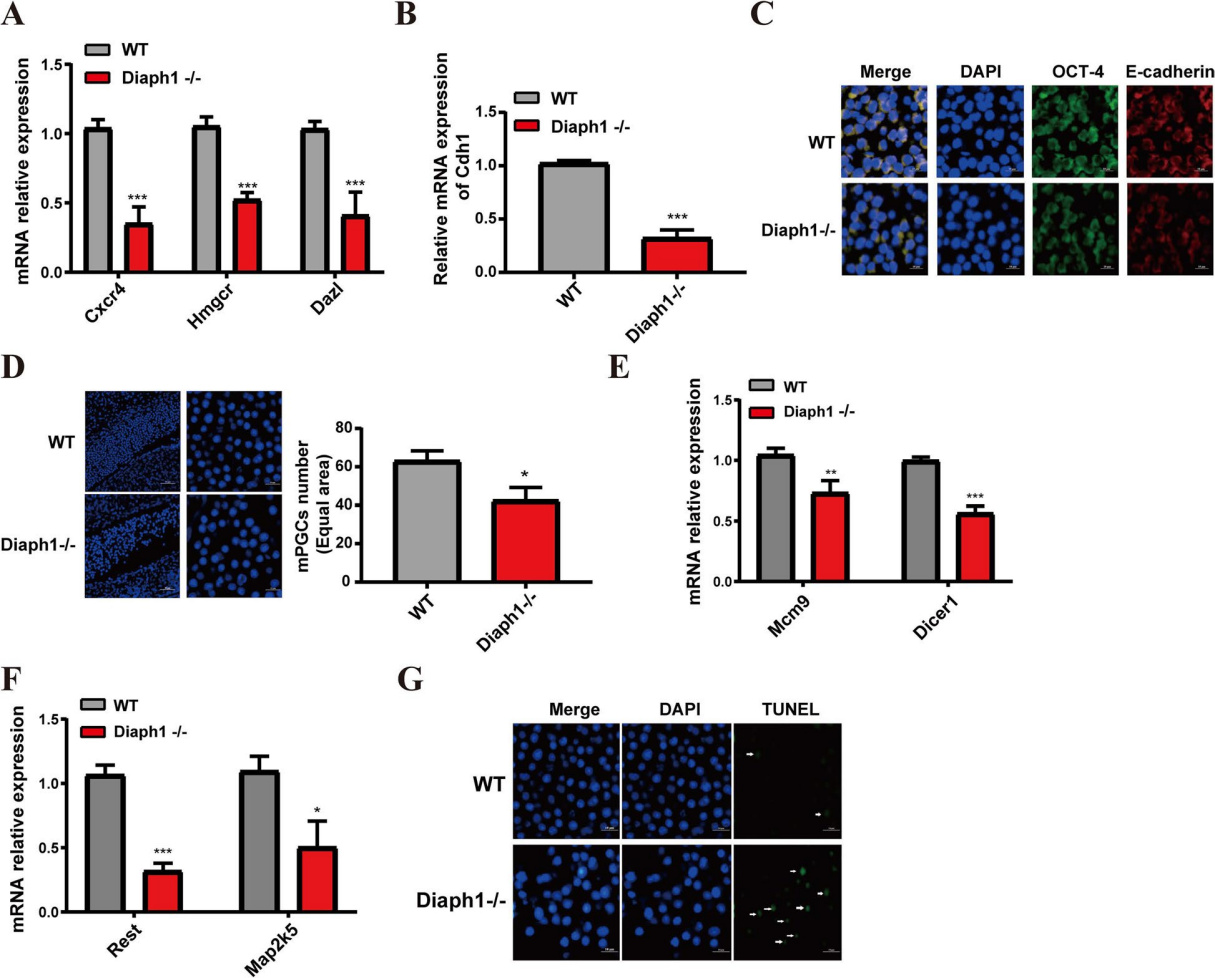
Knockout of *Diaph1* suppresses cell proliferation and promotes cell apoptosis. **A**, **B** The expression of migration-related genes (**A**) and cell adhesion gene (**B**) by qRT-PCR. **C** Immunofluorescence staining of E‐cadherin was indicated. **D** *Diaph1* Knockout reduced the number of PGCs. **E**, **F** Knockout of *Diaph1* decreased the expression of proliferation-related genes (**E**) and cell survival-related genes (**F**). **G** Representative immunofluorescence staining of TUNEL (green) and DAPI (blue)

To demonstrate the above speculation, we used a CRISPR-Cas9 system to generate *Diaph1* knockout mice (Additional file 1: Fig. S1A). Subsequent qRT-PCR and western blot analysis confirmed the absence of DIAPH1 expression in the knockout mice (Additional file 1: Fig. S1B-C). Moreover, we examined the expression of migration-related genes (*Cxcr4*, *Hmgcr*, and *Dazl*) and cell adhesion gene (*Cdh1*) using qRT-PCR. The results indicated a decrease in the expression of migration-related and cell adhesion genes in *Diaph1*^−/−^ PGCs compared to wild-type (WT) PGCs (Fig. 3A-3B). Immunofluorescence assay also revealed reduced expression of E-cadherin fluorescence in *Diaph1*^−/−^ PGCs (Fig. 3C). In addition, the number of PGCs in the *Diaph1*^−/−^ embryos at E12.5 was significantly lower than that of WT embryos (Fig. 3D). The expression levels of proliferation-related genes (*Dicer1*, *Mcm9*) and cell survival-related genes (*Map2k5*, *Rest*) were significantly reduced in *Diaph1*^−/−^ PGCs compared to WT PGCs, as confirmed by qRT-PCR analysis (Fig. 3E-3F). TUNEL staining of sections from E12.5 embryos showed a notable increase in the proportion of apoptotic PGCs in *Diaph1*^−/−^ mice compared to WT (Fig. 3G). These findings suggest that knockdown of *Diaph1* inhibits PGC proliferation and induces PGC apoptosis.

### *Diaph1* knockout suppresses the gonadal growth and fecundity of mouse

**Fig. 4:**
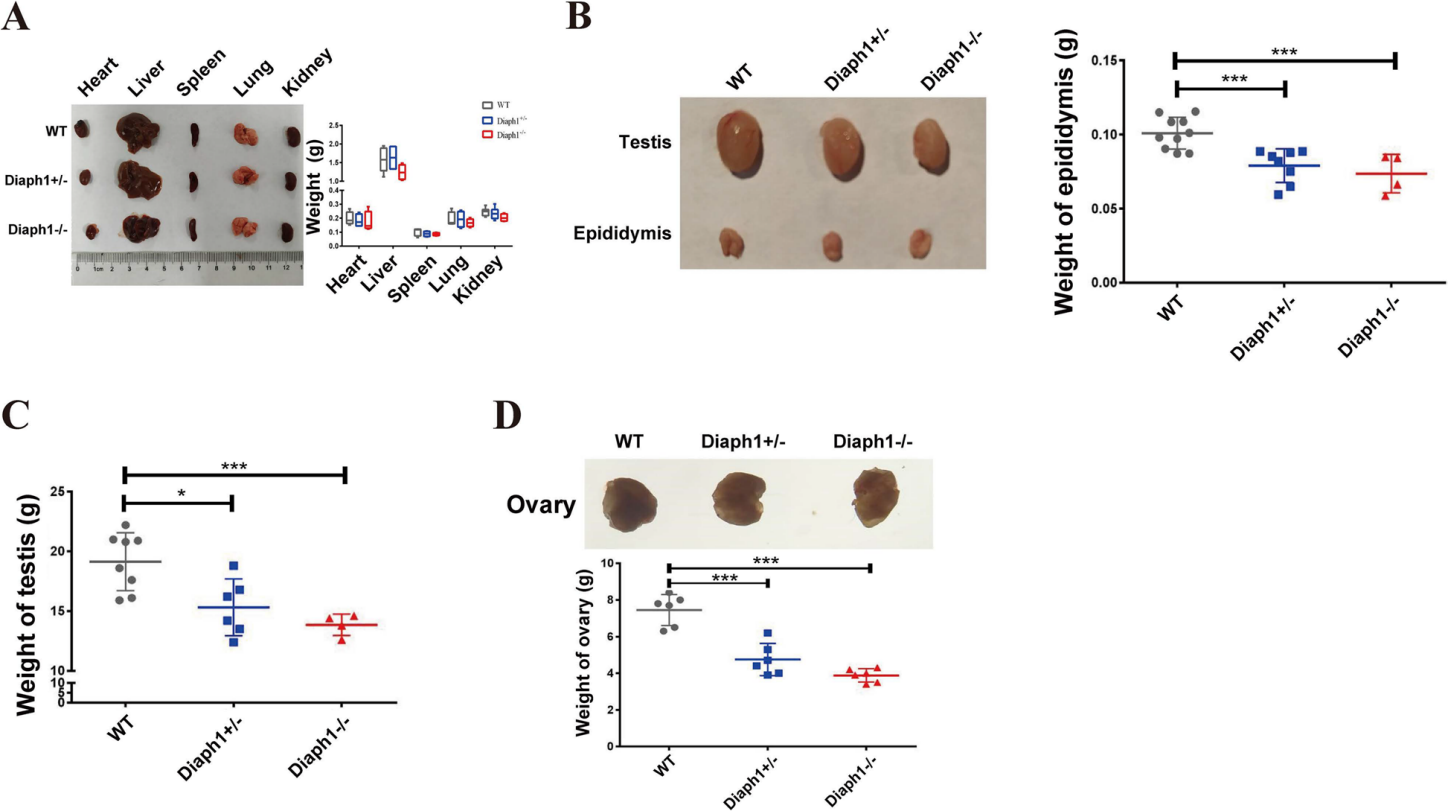
Loss of *Diaph1* leads to decreased the weight of testis and ovary in mice. **A** The weight of heart, liver, spleen, lung and kidney in normal and *Diaph1* knockout groups. **B**-**D** Relative weights of epididymis (**B**), testis (**C**), and ovary (**D**)

Breeding and offspring analysis were conducted in both the WT mouse and the Diaph1 knockout mouse. The results revealed that the genotypic distribution of neonatal litters in heterozygous mice (*Diaph1*^±^) mated with WT mice adhered to Mendel's law. However, there was a discrepancy with Mendel's law when considering the genotypes of offspring resulting from heterozygous mattings (*Diaph1*^±^ x *Diaph1*^±^) (Additional file 2: Table 3). Additionally, the number of offspring resulting from heterozygous mattings was decreased in comparison to heterozygous mattings with WT mice (Additional file 2: Table 3). Furthermore, the heart, liver, spleen, lung, kidney, testis, epididymis, and ovary tissues were weighed in WT, *Diaph1*^±^, and *Diaph1*^−/−^ mice of similar age and weight. The findings demonstrated no significant differences in the heart, liver, spleen, lung, and kidney tissues among the three groups (Fig. 4A). However, the weights of the testis, epididymis, and ovary in both *Diaph1*^±^ and *Diaph1*^−/−^ mice were significantly decreased compared to WT mice (Fig. 4B-D). These results suggestthat *Diaph1* may be involved in testicular development and ovarian development.

### Knocking out *Diaph1* inhibit mouse testicular development

**Fig. 5:**
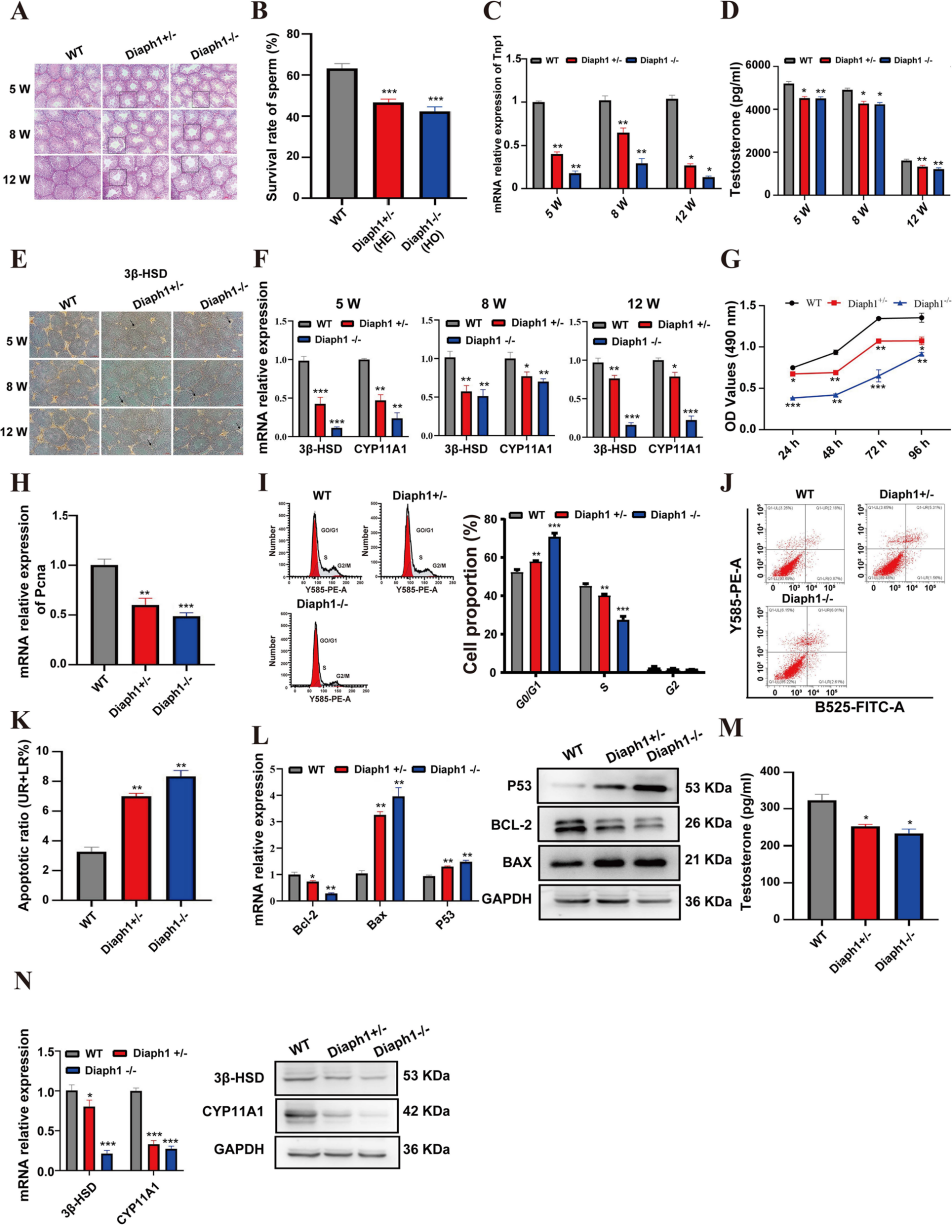
Impact of *Diaph1* knockout on the testis development. **A** HE stain of testis tissues in wild-type and Diaph1knockout mice. **B** The survival rates of sperm in *Diaph1* knockout and WT mice. **C** Verification of *Tnp1* expression by qRT-PCR. **D** Secretion levels of testosterone in mouse serum. **E** Immunohistochemical staining of 3β-HSD expression in testicular tissue. **F** Gene expressions of 3β-HSD and CYP11A1 in testicular tissue. **G** CCK8 assays were performed to assess Leydig cell proliferation. **H** Verification of *Pcna* expression by qRT-PCR. **I** *Diaph1* knockout arrests the cycle progression of Leydig cells. **J**, **K** Knockout of *Diaph1* increased the apoptosis of Leydig cells. **L** The expression of BCL-2, BAX, and P53 was verified by qRT‒PCR and western blot. **M** Secretion levels of testosterone in Leydig cell. **N** The expression of f 3β-HSD and CYP11A1 was verified by qRT‒PCR and western blot

To confirm the above speculation, testicular tissue from male mice of different genotypes (WT, *Diaph1*^±^, *Diaph1*^−/−^) at various ages (5W, 8W, 12W) were subjected to Hematoxylin–Eosin (HE) staining. The results revealed that WT mice exhibited normal testicular structure with intact seminiferous tubules, whereas *Diaph1*^±^ and *Diaph1*^−/−^ male mice displayed abnormal testicular development, characterized by irregularly shaped seminiferous tubules and an enlarged internal cavity area (The rectangular box) (Fig. 5A). Moreover, the sperm parameters of *Diaph1*^±^ and *Diaph1*^−/−^ male mice demonstrated a significant reduction in sperm viability compared to WT male mice (Fig. 5B). The expression of *Tnp1*, a gene associated with spermatogenic function, was evaluated in testicular tissue using qRT-PCR. The results showed a significant decrease in *Tnp1* expression in the testes of *Diaph1*^±^ and *Diaph1*^−/−^ male mice compared to WT male mice (Fig. 5C). Furthermore, the levels of serum testosterone were markedly decreased in *Diaph1*^±^ and Diaph1^−/−^ male mice, as determined by enzyme-linked immunosorbent assay (ELISA) (Fig. 5D). Therefore, we further investigated the expression of enzymes correlated with testosterone synthesis in testis tissues. Immunohistochemical staining for 3β-hydroxysteroid dehydrogenase (3β-HSD) demonstrated decreased staining intensity in *Diaph1*^±^ and *Diaph1*^−/−^ male mice compared to WT mice (Fig. 5E). The expression of 3β-HSD and cytochrome P450 family 11 subfamily A member 1 (CYP11A1) was further assessed using qRT-PCR. The results showed a significant decrease in the expression of *3β-hsd* and *Cyp11a1* in the testes of *Diaph1*^±^ and *Diaph1*^−/−^ mice compared to WT mice (Fig. 5F).

Testosterone is primarily produced in the Leydig cells of the male testes, which have crucial roles in testicular development [29, 30]. Therefore, we conducted further investigations into the effects of *Diaph1* knockout on Leydig cell development in mice. Initially, Leydig cells were individually isolated from male mice of different genotypes (WT, *Diaph1*^±^, *Diaph1*^−/−^), and their proliferation rates were determined. The results from the CCK8 assay demonstrated that the proliferation of Leydig cells was significantly reduced in the presence of *Diaph1* knockout compared to the control group (Fig. 5G). QRT-PCR analysis revealed a significant downregulation of the proliferation-related gene *Pcna* in Leydig cells upon *Diaph1* knockout, compared to the control group (Fig. 5H). To elucidate the underlying reason for the inhibition of Leydig cell proliferation caused by *Diaph1* knockout, we examined its effects on cell cycle progression. As illustrated in Fig. 5I, *Diaph1* knockout led to a substantial increase in the proportion of cells in the G0/G1 phase in Leydig cells compared to control Leydig cells. Moreover, flow cytometry results demonstrated an elevated rate of apoptosis in *Diaph1* knockout Leydig cells compared to the control group cells (Fig. 5J-K). These findings were consistent with the downregulation of BCL2 and upregulation of BAX and P53 (Fig. 5L), suggesting the involvement of *Diaph1* knockout in the regulation of cell cycle and apoptosis in Leydig cells. Furthermore, we assessed the levels of testosterone in Leydig cells. ELISA analyses revealed a significant decrease in testosterone levels in *Diaph1* knockout Leydig cells compared to the control group (Fig. 5M). Subsequently, we examined the expression of enzymes associated with testosterone synthesis in *Diaph1* knockout Leydig cells. qRT-PCR and Western blotting results demonstrated a significant decrease in the expression of 3β-HSD and CYP11A1 mRNA and protein in *Diaph1* knockout Leydig cells, relative to WT Leydig cells (Fig. 5N). These results indicate that knocking out *Diaph1* affects mouse testicular development.

### Knocking out *Diaph1* inhibit mouse ovarian development

**Fig. 6:**
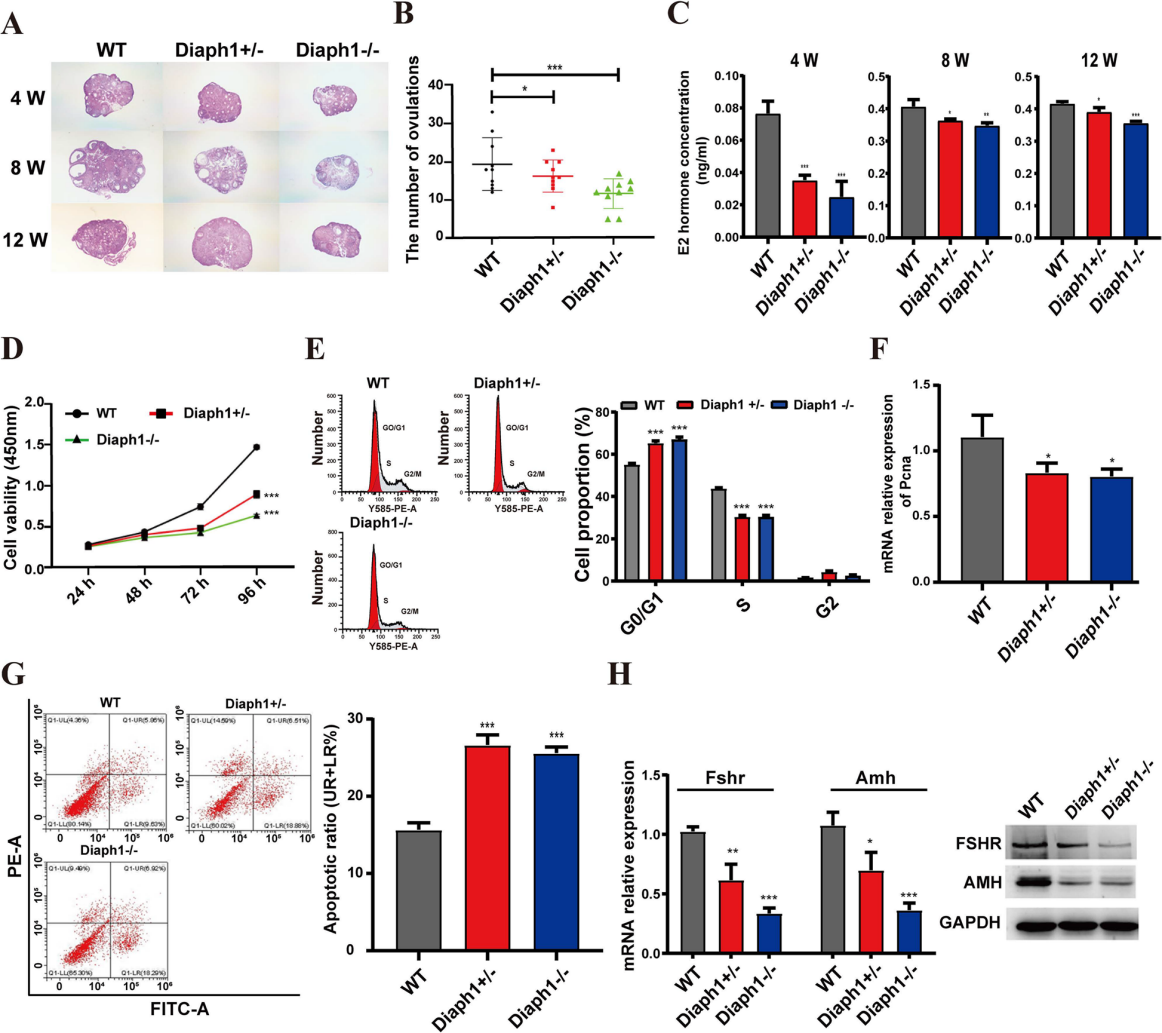
Impact of *Diaph1* knockout on the ovary development. **A** HE stain of ovarian tissues in wild-type and *Diaph1* knockout mice. **B** Ovulation number of *Diaph1* knockout mice. **C** Serum estradiol levels. **D** CCK8 assays were performed to assess granulosa cell proliferation. **E** Flow cytometric analysis of the cell cycle. **F** The expression of *Pcna* was verified by qRT‒PCR. **G** Flow cytometry analysis of apoptosis in ovarian granulosa cells. **H**, **I** The expression of FSHR and AMH was verified by qRT‒PCR (**H**) and western blot (**I**)

Previous data suggest that knocking out *Diaph1* may inhibit ovarian development (Fig. 4D). To confirm this speculation, HE staining was performed on ovarian tissues from *Diaph1*^±^, *Diaph1*^−/−^, and WT female mice at 4 weeks, 8 weeks, and 12 weeks of age. The results demonstrated an apparent decrease in follicle development in *Diaph1*^±^ and *Diaph1*^−/−^ ovarian tissue compared to WT ovarian tissue (Fig. 6A). Superovulation was performed on female mice of the WT, *Diaph1*^±^, and *Diaph1*^−/−^ genotypes. The results demonstrated a significant decrease in the number of ovulations in *Diaph1*^±^ and *Diaph1*^−/−^ mice compared to WT mice (Fig. 6B). Moreover, ELISA results demonstrated a significant decrease in serum estradiol levels in *Diaph1*^±^ and *Diaph1*^−/−^ mice compared to WT mice (Fig. 6C), while other hormone levels remained unaffected (Additional file 1: Fig. S1D-F).

Estradiol is primarily produced by the granulosa cells of the ovaries [31]. Therefore, we speculated whether *Diaph1* affect ovarian development through their effect on granulosa cells. To confirm our speculation, we isolated granulosa cells individually from female mice of three groups: WT, *Diaph1*^±^, and *Diaph1*^−/−^. Results from the CCK8 assay revealed a significant decrease in cell proliferation in the *Diaph1*^±^ and *Diaph1*^−/−^ granulosa cells compared to the WT granulosa cells (Fig. 6D). Flow cytometry analysis revealed that *Diaph1* knockout led to a significant increase in the proportion of G0/G1 phase cells and a notable decrease in the S phase in granulosa cells, as compared to control granulosa cells (Fig. 6E). Furthermore, the knockout of *Diaph1* significantly inhibited the expression of the proliferation-related gene *Pcna* (Fig. 6F) and promoted apoptosis of granulosa cells (Fig. 6G). We also examined the expression of granulosa cell markers (FSHR and AMH) and found that both mRNA and protein expression of FSHR and AMH were significantly decreased in *Diaph1* knockout granulosa cells compared to WT granulosa cells (Fig. 6H). These results indicate that the knockout of *Diaph1* inhibits ovarian development in mice.

## Discussion

Primordial germ cells (PGCs), the precursors for the gametes, undergo specification, migration, and proliferation during embryonic development [32]. Previous studies have primarily focused on exploring the role of chemokines/chemokines receptor [33–35], adherence factors [36] and integrins [37] relation to PGC migration. However, the regulatory role of formin family proteins during migration of PGCs remains largely understudied. The formin family proteins are considered essential factors for promoting actin polymerization [38], and remodeling of the actin cytoskeleton, which is crucial in both the developmental processes and the maintenance of tissue homeostasis [39]. In addition, the formin family proteins play important roles in spermatogenesis [40, 41]. The aberrant function and expression of formin have been directly implicated in maladies as diverse as deafness [42], cancer [43], and fertility defects [44]. Here, based on chromatin property data (DNase-seq) and gene expression data (RNA-seq), the differentially expressed gene *Diaph1* (also known as *mDia1*), a member of the formin family, was screened during mouse PGC (mPGC) migration, suggesting an important role for the *Diaph1* during mPGC migration.

*Diaph1* is involved in a variety of cell functions including cell morphology, differentiation, adhesion, and migration [45–48], and thus regulating physiological state, shape function, and pathological mechanisms of cells [49, 50]. However, previous research on the role of *Diaph1* in cell migration mainly focused on tumor cells and nerve cells. For example, *Diaph1* knockdown suppress migration and reduces the expression of matrix metallopeptidase MMP2 and MMP9 in human glioma cells [28]. *Diaph1* has also been implicated in neural migration in brain development [47]. In this study, *Diaph1* was significantly differentially expressed during mPGC migration. Knockout of *Diaph1* suppressed mPGC proliferation and promoted apoptosis of PGCs. Furthermore, knockout of *Diaph1* inhibited the expression of proliferation-related genes (*Dicer1*, *Mcm9*), adhesion-related factors (*E-cadherin*, *Cdh1*), and migration-related genes (*Cxcr4*, *Hmgcr*, *Dazl*), indicating that knockout of *Diaph1* impairs PGC proliferation and survival.

Previous studies have found that *Diaph1* knockout mice show marked reductions in the ratios of heart weight to tibia length, cross-sectional area of cardiomyocytes, left ventricular wall thickness, and expression levels of hypertrophic-specific genes [51]. However, the precise involvement of *Diaph1* in gonadal development remains ambiguous, warranting further investigation. In this study, we found that testis weight and sperm number significantly decreased in *Diaph1* knockout mice, and *Diaph1* knockout resulted in reduced levels of serum testosterone, which plays a crucial role in male testes development, as well as in pubertal virilization and the initiation of spermatogenesis [52, 53]. Testosterone is primarily produced by Leydig cells in the testicles through the activation of a series of steroidogenic enzymes. These enzymes include cytochrome P450 side-chain cleavage enzyme CYP11A1 and 3β-hydroxysteroid dehydrogenase (3β-HSD) [54, 55]. The former converts cholesterol into pregnenolone, while the latter converts pregnenolone into testosterone in the smooth endoplasmic reticulum [56]. Alterations of the expression of CYP11A1 and 3β-HSD can affect the testosterone synthesis [56]. Here, the mRNA and protein expression levels of CYP11A1 and 3β-HSD were decreased following *Diaph1* knockout in the Leydig cells. It is important to note that testosterone production is influenced not only by the expression levels of CYP11A1 and 3β-HSD but also by the number of Leydig cells [57, 58]. We found that *Diaph1* knockout inhibits the proliferation of Leydig cells and induces their apoptosis. These data indicate that knocking out *Diaph1* can disrupt the proliferation of mouse Leydig cells and the expression of CYP11A1 and 3β-HSD proteins in Leydig cells, leading to impaired testosterone secretion and maintenance of germ cells, thereby affecting reproductive function.

It has been demonstrated that silencing *Diaph1* impaired mitochondrial trafficking and cortisol biosynthesis and concomitantly increased the secretion of adrenal androgens [59]. In addition, the plasma DIAPH1 levels were found to be associated with sex hormones (estrogen, progesterone, and luteinizing hormone (LH)/follicle-stimulating hormone (FSH) in patients with polycystic ovary syndrome [60]. Currently, the impact of *Diaph1* on sex hormone synthesis is primarily focused on hormone level detection, while further research is needed to understand its specific regulatory mechanisms. In this study, we observed a significant decrease in developing follicle counts in the ovaries of *Diaph1* knockout mice, as well as a reduction in serum estradiol levels upon *Diaph1* knockout. Further research found that *Diaph1* knockout reduces the expression of FSH receptor (FSHR) in granulosa cells, although no changes in serum FSH levels were observed in *Diaph1* knockout mice. Furthermore, *Diaph1* knockout inhibits the proliferation and promotes apoptosis of granulosa cells. Estradiol is locally produced by granulosa cells of developing antral follicles upon FSH stimulation to strengthen follicular growth and maturation [61]. FSH activates the FSHR in granulosa cells, thereby inducing follicular differentiation, growth, and estradiol production [62, 63]. These results suggest that *Diaph1* regulate the expression of FSHR, reduce the sensitivity of granulosa cells to FSH, inhibit the proliferation of granulosa cells, thereby affecting the secretion of estradiol.

## Conclusions

In summary, this study demonstrated the effects of *Diaph1* knockout on the migration of mouse PGCs and the synthesis of sex hormones. The deficiency of *Diaph1* compromises the survival and proliferation of PGCs. Additionally, the knockout of *Diaph1* could interfere with the expression of sex hormone regulatory factors, thereby causing the disorder of sex hormone secretion in the body and the maintenance of germ cell, resulting in reproductive function damage. These results laid a solid foundation for further research on the molecular mechanism of PGC migration and gonadal development, and have promising implications for reproductive medicine.

### Supplementary Information

Additional file 1: Fig S1. A Schematic experimental design illustrating the Diaph1 knockout in mice. B-C The expression of DIAPH1 was verified by qRT‒PCR (B) and western blot (C). D-F: Secretion levels of LH (D), FSH (E), and Androgen (F) hormone in mouse serum. Bar = mean ± SD. ***p < 0.001.

Additional file 2: Table 1. PCR identification primers for *Diaph1* knockout mice. Table 2. Primer sequences of genes. Table 3. Reproduction data for *Diaph1* knockout mouse.

Additional file 3. Western blot raw data.

## Data Availability

No datasets were generated or analysed during the current study.

## Abbreviations

PGCs: Primordial germ cells
DIAPH1: Diaphanous related formin 1
E: Embryonic day
FSH: Follicle-stimulating hormone
FSHR: FSH receptor
E2: Estradiol
LH: Luteinizing hormone
CYP11A1: Cytochrome P450 family 11 subfamily A member 1
3β-HSD: 3β-Hydroxysteroid dehydrogenase
qRT‒PCR: Quantitative real-time polymerase chain reaction
