# Supplementary figures and images for: Diaph1 knockout inhibits mouse primordial germ cell proliferation and affects gonadal development
Source: Reprod Biol Endocrinol. 2024 Jul 15;22:82. doi: 10.1186/s12958-024-01257-z (PMC11247884; doi:10.1186/s12958-024-01257-z)

**A**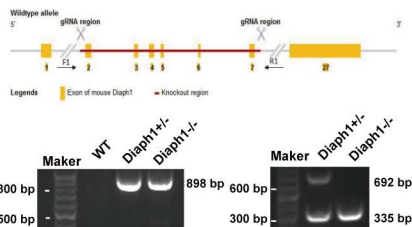**B**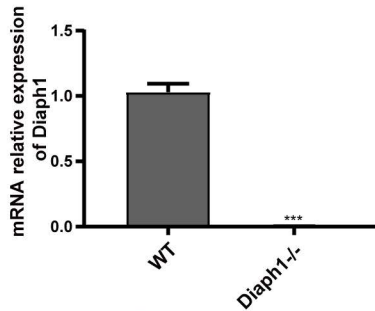**C**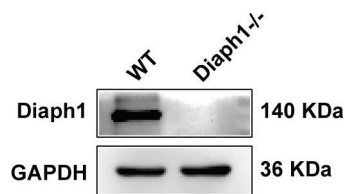**D**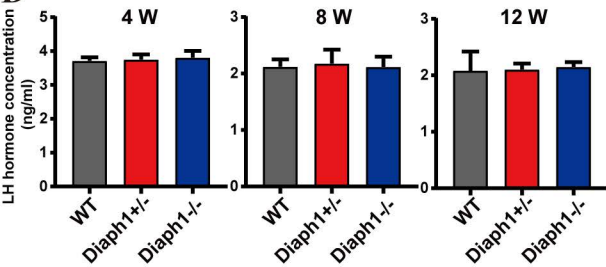**E**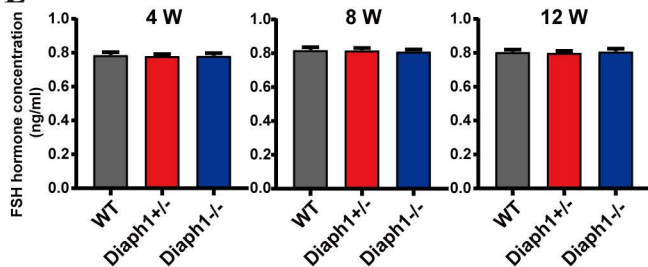**F**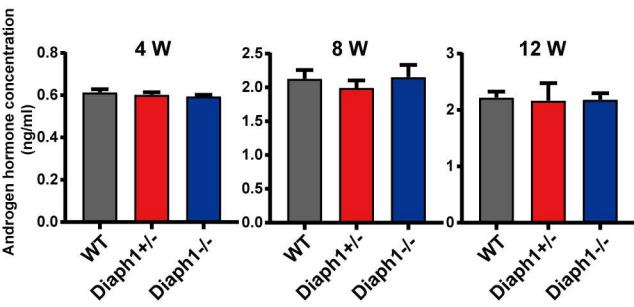

Supplement: Supplementary file 1 — Additional file 1: Fig S1. A Schematic experimental design illustrating the Diaph1 knockout in mice. B-C The expression of DIAPH1 was verified by qRT‒PCR (B) and western blot (C). D-F: Secretion levels of LH (D), FSH (E), and Androgen (F) hormone in mouse serum. Bar = mean ± SD. ***p < 0.001. [file 12958_2024_1257_MOESM1_ESM.pdf]

# Additional file 3

## Western blot raw data

Fig. 5L

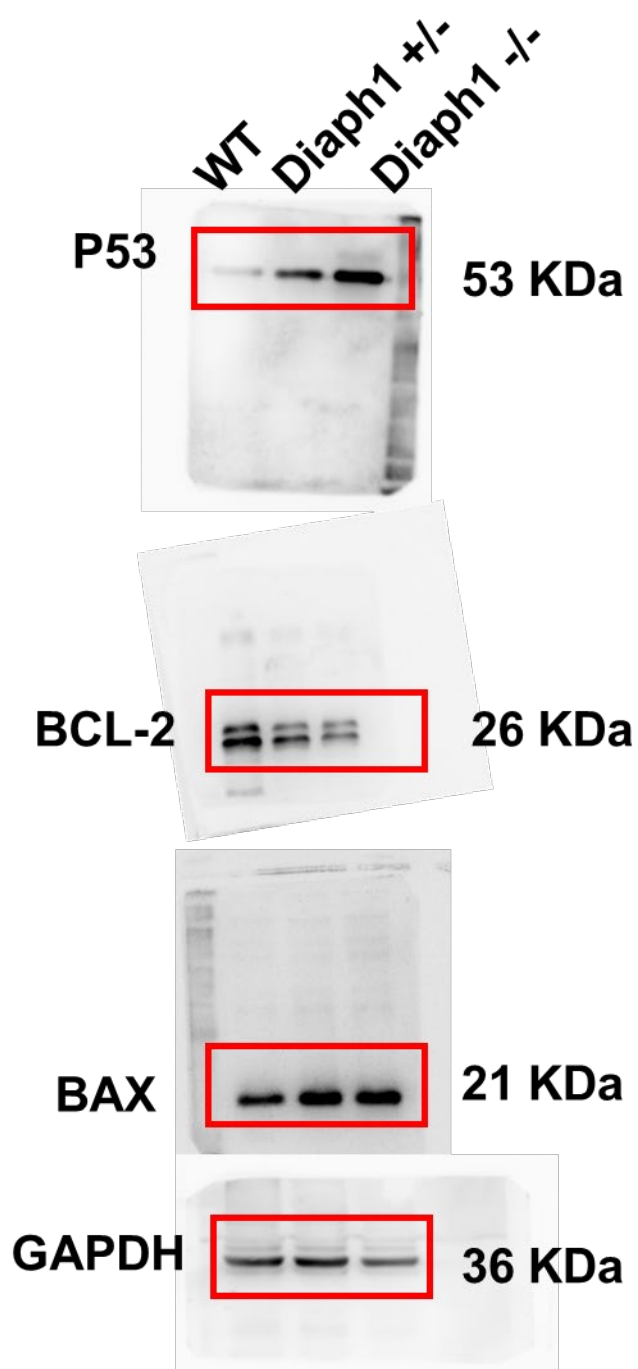

**Fig. 5N**

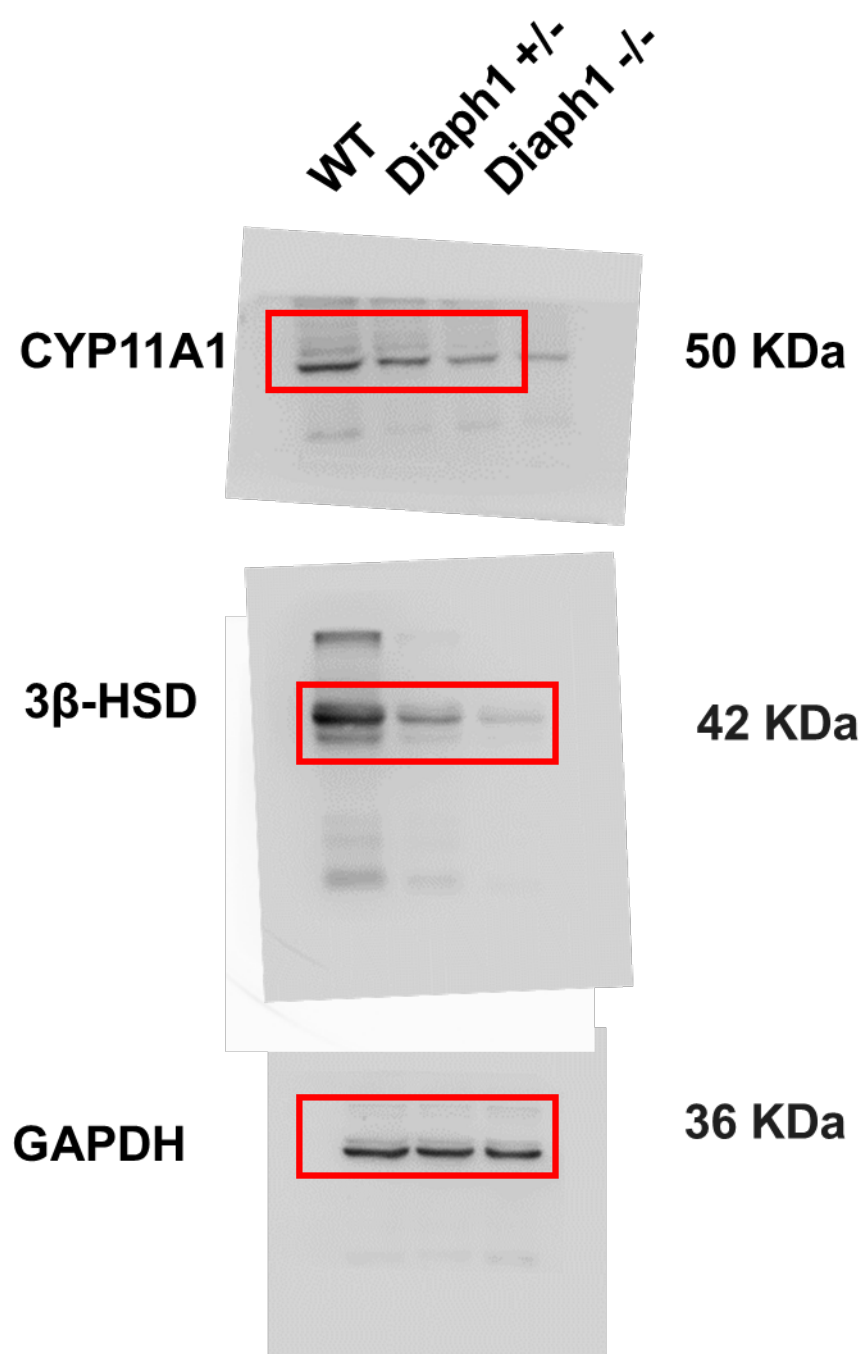

**Fig. 6H**

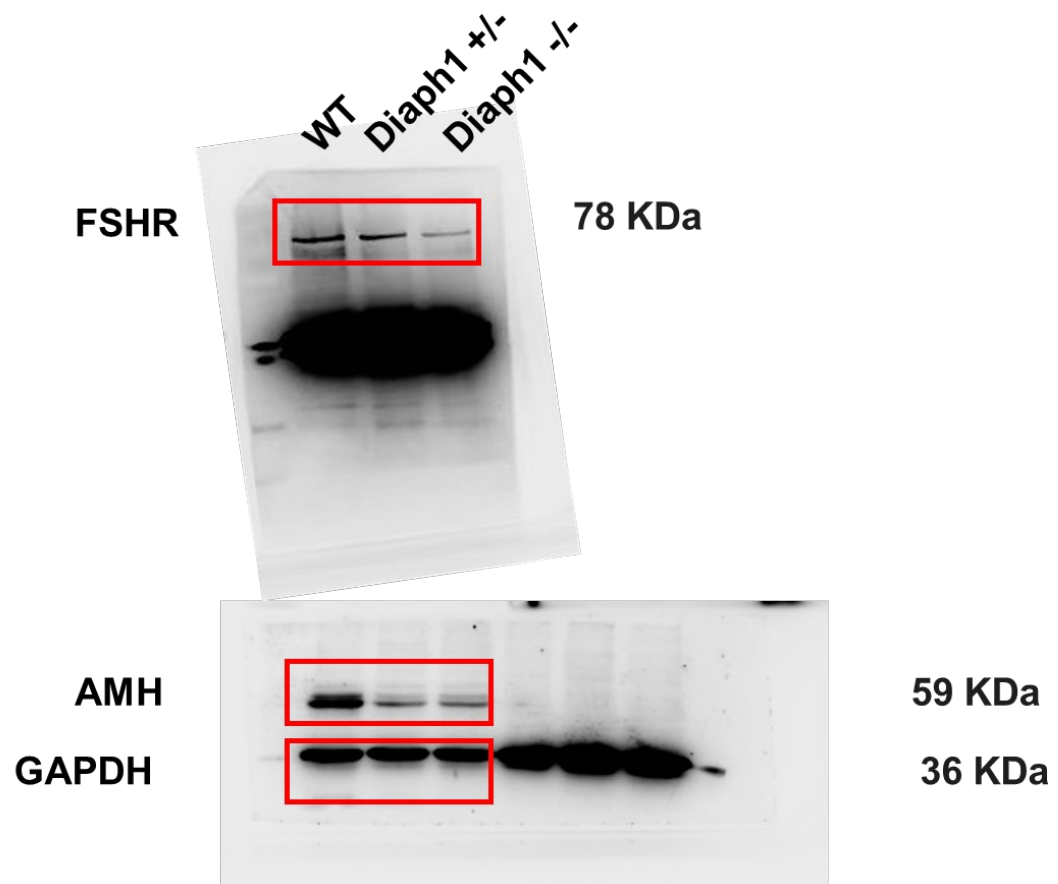

**Additional file 3: Fig. 1C**

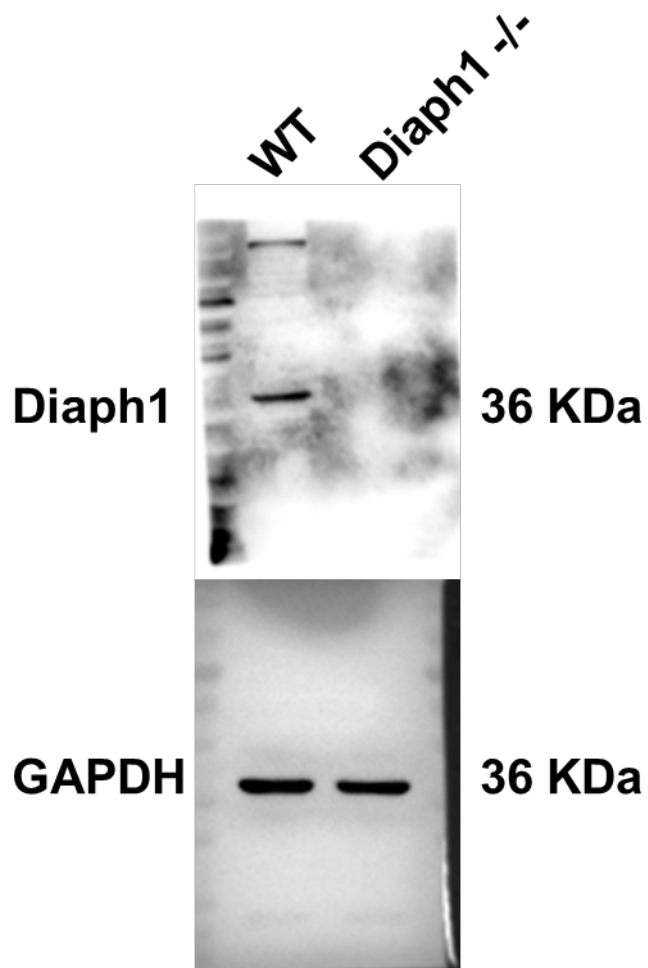

Supplement: Supplementary file 3 — Additional file 3. Western blot raw data. [file 12958_2024_1257_MOESM3_ESM.pdf]
