# Supplementary material for: Diaph1 knockout inhibits mouse primordial germ cell proliferation and affects gonadal development
Source: Reprod Biol Endocrinol. 2024 Jul 15;22:82. doi: 10.1186/s12958-024-01257-z (PMC11247884; doi:10.1186/s12958-024-01257-z)
Supplement: Supplementary file 2 — Additional file 2: Table 1. PCR identification primers for Diaph1 knockout mice. Table 2. Primer sequences of genes. Table 3. Reproduction data for Diaph1 knockout mouse. [file 12958_2024_1257_MOESM2_ESM.docx]

**Table 1. PCR identification primers for *Diaph1* knockout mice**

| Primers | Sequence (5’-3’) |
| --- | --- |
| *Diaph1*-F1 | GCTCTCCACTCTCTCTATCACCAAG |
| *Diaph1*-R1 | TAACCTGGTCTATGAGTTTGAGGTC |
| *Diaph1*-R2 | GCATGACACCTACACAGCAAAGAAT |
| *Diaph1*-Internal control F | CATGCCAATGGTTCACTCTAAGGT |
| *Diaph1*-Internal control R | TCTCTATGTCCCAAAGTGCAGACAC |

**Table 2. Primer sequences of genes**

| Genes | Sequences (5’-3’) | Genes | Sequences (5’-3’) |
| --- | --- | --- | --- |
| *Gapdh* | F: TGACGTGCCGCCTGGAGAAA CTTCAACAGCGACACTCACTCT | *Nanog* | F: CAGGTGTTTGAGGGTAGCTC |
|  | R: AGTGTAGCCCAAGATGCCCTTCAG |  | R: CGGTTCATCATGGTACAGTC |
| *Diaph1* | F: TAGTGGGCCTTCCCTAGGTC | *Oct4* | F: CAGACCACCATCTGTCGCTTC |
|  | R: CTCCCAATGCCACAGCACTA CGATGATGCAAGTCGCAATTАC |  | R: CTCACACGGTTCTCAATGCTAGTTC |
| *Cyp11a1* | F: CCAGTGTCCCCATGCTCAAC | *Mvh* | F: GTTTGCATCTGTTGACACGAGGA |
|  | R: TGCATGGTCCTTCCAGGTCT |  | R: CAACTGGATTGGGAGCTTGTGA |
| *3β-HSD* | F: TATTCTCGGTTGTACGGGCAA | *Dazl* | F: GCCTCCAACCATGATGAATCCTA |
|  | R: GTGCTACCTGTCAGTGTGACC |  | R: TCCAGTGATGACCTGAACTGGTG |
| *Pcna* | F: AAAGATGCCGTCGGGTGAAT | *Fkbp6* | F: CCTCGGCTGATGAAACTTGGA |
|  | R: TGGTTACCGCCTCCTCTTCT |  | R: CATAGGCTGGCTTGAACAGGAAC |
| *Cdh1* | F: CAGGTCTCCTCATGGCTTTGC | *Spo11* | F: TGCTGGCAACTTGAGATACATGG |
|  | R: CTTCCGAAAAGAAGGCTGTCC |  | R: TGAATGTTAGTCGGCACAGCAGTAG |
| *Mcm9* | F: GGTCAGGTGTTTGAGTCCTATG | *Cxcr4* | F: AGCCTGTGGATGGTGGTGTTTC |
|  | R: GCATTAACCACAACCGGGTAG |  | R: CCTTGCTTGATGACTCCCAAAAG |
| *Dicer1* | F: GGTCCTTTCTTTGGACTGCCA | *Hmgcr* | F: AGCTTGCCCGAATTGTATGTG |
|  | R: GCGATGAACGTCTTCCCTGA |  | R: TCTGTTGTGAACCATGTGACTTC |
| *Rest* | F: CCCCTTCGCAACCACTTCTC | *Mov10l1* | F: TGCGACTTCATGAGAGCAAGG |
|  | R: CTTTGAGGTCAGCCGACTCT |  | R: CATCTCTGCAATACGGTTTGATGG |
| *Map2k5* | F: AAGCAGCCCAAGGAGAGAC | *4930432*  *K21Riken* | F: CAGAGCAACTTCCATTACTGCTGTG |
|  | R: GAACTGCACGATGAATGGGTG |  | R: AGGAGCAGGTTCTGAGCTGTCTTC |

**Table 3. Reproduction data for *Diaph1* knockout mouse**

| Mating information | WT*  Sex ratio  Male: female | HE*  Sex ratio  Male: female | HO*  Sex ratio  Male: female | Number of progeny genotypes | | | Average litter size |
| --- | --- | --- | --- | --- | --- | --- | --- |
|  |  |  |  | WT | HE | HO |  |
| WT×WT | 50:52 | - | - | 102 |  |  | 9±3 |
| WT×HE | 23:27 | 29:27 | - | 50 | 56 | - | 9±1 |
| WT×HO | - | 8:7 | - | - | 75 | - | 6±2 |
| HE×HE | 59:56 | 73:78 | 27:28 | 115 | 151 | 55 | 7±2 |
| HE×HO | - | 39:44 | 27:31 | - | 83 | 58 | 6±2 |
| HO×HO | - | - | 10:9 | - | - | 38 | 3±3 |

* WT: Wild type; HE: Heterozygote; HO: Homozygote.
